# Antimicrobial-drug Susceptibility of Human and Animal *Salmonella* Typhimurium, Minnesota, 1997–2003

**DOI:** 10.3201/eid1112.050158

**Published:** 2005-12

**Authors:** Stephanie D. Wedel, Jeffrey B. Bender, Fe T. Leano, David J. Boxrud, Craig Hedberg, Kirk E. Smith

**Affiliations:** * Minnesota Department of Health, Minneapolis, Minnesota, USA; † University of Minnesota College of Veterinary Medicine, St. Paul, Minnesota, USA; ‡ University of Minnesota School of Public Health, Minneapolis, Minnesota, USA

**Keywords:** Salmonella Typhimurium, antimicrobial resistance, pulsed-field gel electrophoresis, human, animal, research

## Abstract

We compared antimicrobial resistance phenotypes and pulsed-field gel electrophoresis (PFGE) subtypes of 1,028 human and 716 animal *Salmonella enterica* serotype Typhimurium isolates from Minnesota from 1997 to 2003. Overall, 29% of human isolates were multidrug resistant. Predominant phenotypes included resistance to ampicillin, chloramphenicol or kanamycin, streptomycin, sulfisoxazole, and tetracycline (ACSSuT or AKSSuT). Most human multidrug-resistant isolates belonged to PFGE clonal group A, characterized by ACSSuT resistance (64%), or clonal group B, characterized by AKSSuT resistance (19%). Most animal isolates were from cattle (n = 358) or swine (n = 251). Eighty-one percent were multidrug resistant; of these, 54% were at least resistance phenotype ACSSuT, and 43% were at least AKSSuT. More than 80% of multidrug-resistant isolates had a clonal group A or B subtype. Resistance to ceftriaxone and nalidixic acid increased, primarily among clonal group A/ACSSuT isolates. Clonal group B/AKSSuT isolates decreased over time. These data support the hypothesis that food animals are the primary reservoir of multidrug-resistant *S*. Typhimurium.

## Abstract 2

Food animals are likely the primary reservoir of resistant *S.* Typhimurium.

Nontyphoidal salmonellae are a leading cause of acute gastroenteritis in the United States (*1*). *Salmonella enterica* serotype Typhimurium is the most common serotype isolated from humans (*2*). In the 1990s, multidrug-resistant (MDR) *S*. Typhimurium definitive phage type 104 (DT104) emerged in the United States; most isolates were resistant to ampicillin, chloramphenicol, streptomycin, sulfisoxazole, and tetracycline (resistance phenotype [R-type] ACSSuT) (*3*). *S*. Typhimurium R-type AKSSuT (with resistance to kanamycin) has also recently emerged in the United States (*4*). Several studies have documented adverse health effects due to the increasing resistance observed in *S*. Typhimurium (*5**–**9*). These effects include an increased risk for infection with *S*. Typhimurium (*5*), increased risk for bloodstream infection (*6*), increased risk for hospitalization (*6**,**7*), treatment failures (*8*), and increased risk for death (*9*).

MDR *S*. Typhimurium strains have been well documented in food animals, as have MDR *S*. Typhimurium outbreaks in humans from animal contact or foods of animal origin (*8**,**10**–**17*). However, contemporaneous parallel data on resistance in human and animal *S*. Typhimurium isolates in the United States are limited (*18*), and an advisory panel has called for linking surveillance for bacterial resistance in animals and humans to further evaluate the human health effects of antimicrobial drug use in agriculture (*19*). The objectives of our study were to evaluate antimicrobial resistance and molecular subtyping data from all human clinical *S*. Typhimurium isolates received through statewide, population-based, active laboratory surveillance in Minnesota and to compare the human isolates to isolates from clinically ill animals in Minnesota identified by the Minnesota Veterinary Diagnostic Laboratory (MVDL).

## Methods

### Human and Animal Isolates

The Minnesota Department of Health (MDH) requires clinical laboratories to submit all *Salmonella* isolates to its public health laboratory as part of active, laboratory-based surveillance. MDH audits clinical laboratories to ensure complete reporting. Human *S*. Typhimurium isolates submitted to MDH from 1997 to 2003 were eligible for this study. Isolates that were part of an identified outbreak were excluded, except for the index case-isolate. Isolates from secondary cases in household clusters and duplicate submissions from the same case also were excluded.

MVDL is a regional laboratory for veterinarians; pertinent diagnostic samples are cultured for *Salmonella* spp. Isolates are sent to the National Veterinary Services Laboratories (Ames, Iowa) for serotyping. Confirmed *S*. Typhimurium isolates are forwarded to MDH. *S*. Typhimurium isolates obtained from diagnostic specimens from sick animals cultured at MVDL from 1997 to 2003 were eligible for this study. Isolates from the same farm with the same pulsed-field gel electrophoresis (PFGE) subtype discovered within 1 year of the initial isolate collection date were excluded. Research animal submission, environmental sample, and non-Minnesota animal isolates were excluded.

### Study Populations

From 1997 to 2003, a total of 4,333 culture-confirmed cases of human salmonellosis were reported in Minnesota. *S*. Typhimurium was the most common serotype; it accounted for 1,193 (28%) cases overall (median 172 cases/year, range 124–201). Of the 1,193 human *S*. Typhimurium case-isolates, 1,028 (86%) were included in this study (Table 1).

**Table 1:** Multidrug-resistance phenotypes of Salmonella enterica serovar Typhimurium isolates from Minnesota residents and animals, 1997–2003*

Resistance phenotype	No. isolates															
	1997		1998		1999		2000		2001		2002		2003		Total	
	167 Hu	150 An	163 Hu	146 An	157 Hu	109 An	152 Hu	67 An	155 Hu	78 An	118 Hu	75 An	116 Hu	91 An	1,028 Hu	716 An
At least pentaresistant	53	132	55	124	50	81	41	49	46	67	22	55	29	72	296	580
AKSSuT	14	76	15	72	11	38	5	18	5	13	2	5	1	10	53	232
ACSSuT	29	18	26	20	26	18	27	17	30	37	12	32	20	38	170	180
At least pentaresistant but not AC or AK	10	38	14	32	13	25	9	14	11	17	8	18	8	24	73	168
ACKSSuT	3	12	4	17	4	4	0	2	0	5	1	2	0	4	12	46
At least ACSSuT + Cr and/or Na†‡	1	3	0	3	0	3	4	1	5	1	2	4	3	8	15	23
At least AKSSuT + Cr and/or Na†‡	1	0	0	0	0	0	0	0	1	0	0	0	0	0	2	0
ACSSuT§ + >2 drugs	1	0	0	3	0	1	0	1	4	2	1	1	4	2	10	10
AKSSuT§ + >2 drugs	3	1	1	0	1	0	0	0	1	0	0	3	1	3	7	7
ACKSSuT + >1 drug	1	20	6	8	0	12	3	5	1	3	1	7	1	9	13	64

*Hu, human; An, animal; A, ampicillin; C, chloramphenicol; K, kanamycin; S, streptomycin; Su, sulfisoxazole; T, tetracycline; Cr, ceftriaxone; Na, nalidixic acid.

†Resistance phenotype ACKSSuT isolates are included as ACSSuT but not AKSSuT.

‡Resistance phenotype ACSSuT accounted for 11 (61%) of 18 human ceftriaxone-resistant isolates, 10 (91%) of 11 human nalidixic acid–resistant isolates, 22 (88%) of 25 animal ceftriaxone-resistant isolates, and 2 (50%) of 4 animal nalidixic acid–resistant isolates. Seven human isolates and 1 animal isolate (from a turkey) were resistant to both ceftriaxone and nalidixic acid; all were multidrug resistant; and 6 of 7 human and the animal isolate were also at least ACSSuT. No isolates were resistant to ciprofloxacin.

§Resistance phenotype ACKSSuT not included as ACSSuT or AKSSuT.

A total of 716 animal isolates were included in this study (median 91/year, range, 67–150) (Table 1). Isolates represented 644 farms and animal owners and 72 of 87 Minnesota counties. Most isolates were of bovine (n = 358, 50%) or porcine (n = 251, 35%) origin. Cattle isolates decreased markedly over time: 106 isolates in 1997, 100 isolates in 1998, 49 isolates in 1999, 31 isolates in 2000, 29 isolates in 2001, 18 isolates in 2002, and 25 isolates in 2003. Conversely, swine isolates increased over time: 32 isolates in 1997, 27 isolates in 1998, 33 isolates in 1999, 22 isolates in 2000, 44 isolates in 2001, 39 isolates in 2002, and 54 isolates in 2003. The remaining isolates included 38 (5%) avian (5 turkey, 1 chicken, 7 unknown, and 25 miscellaneous species), 29 (4%) equine, 21 (3%) feline, 7 (1%) canine, and 12 (2%) other species.

### Isolate Testing

All *S*. Typhimurium isolates (including variant Copenhagen) submitted to MDH were confirmed as *S*. Typhimurium and subtyped by PFGE. PFGE patterns were compared by using BioNumerics software (Applied Maths, Sint-Martens-Latem, Belgium) with the Dice coefficient and a 1% band matching criterion (*20*). Patterns with no visible differences were considered indistinguishable. Subtypes for *S*. Typhimurium at MDH are designated with the prefix "TM" followed by a number (e.g., TM123). PFGE patterns are also submitted to the PulseNet national database. Antimicrobial susceptibility testing was performed with the disc diffusion method and interpretive standards of the National Committee for Clinical and Laboratory Standards (NCCLS) (*21*). Antimicrobial susceptibility was determined for ampicillin (A), chloramphenicol (C), kanamycin (K), streptomycin (S), sulfisoxazole (Su), tetracycline (T), cephalothin (Ct), ceftriaxone (Cr), ciprofloxacin (Cp), gentamicin (G), nalidixic acid (Na), and trimethoprim/sulfamethoxazole (Sxt). The Etest for MIC was performed on isolates with intermediate susceptibility to ceftriaxone by disc diffusion; MICs were interpreted according to NCCLS criteria (*21*). An MIC of 48 μg/mL was considered resistant. Multidrug resistance was defined as resistance to >5 antimicrobial drugs.

PFGE data were analyzed by the first 3 tiers of criteria described by Tenover et al. (0, 1- to 3-, and 4- to 6-band differences) (*22*). Two primary PFGE subtype clusters that accounted for a large proportion of MDR isolates were identified on the basis of a <3-band difference: 1) clonal group A (CGA), composed of subtypes <3 bands different from PFGE subtype TM5b, and 2) clonal group B (CGB), composed of subtypes <3 bands different from PFGE subtype TM54.

### Statistical Analysis

Resistance was analyzed in terms of R-types ACSSuT, AKSSuT, and ACKSSuT. R-type ACKSSuT isolates were included in analyses of "at least R-type ACSSuT" isolates, but not "at least R-type AKSSuT" isolates. Where indicated, ACKSSuT isolates were evaluated independently of ACSSuT. R-types were analyzed in terms of clonal group. The χ^2^ test for trend was used to evaluate resistance trends (EpiInfo 6.04d, Centers for Disease Control and Prevention, Atlanta, GA, USA). Proportions were compared by using the χ^2^ test. Uncorrected p value and exact 95% mid-p limits for the maximum likelihood estimate of the odds ratio (OR) were used. A p value <0.05 was considered significant.

## Results

### Human Isolates

**Figure 1:**
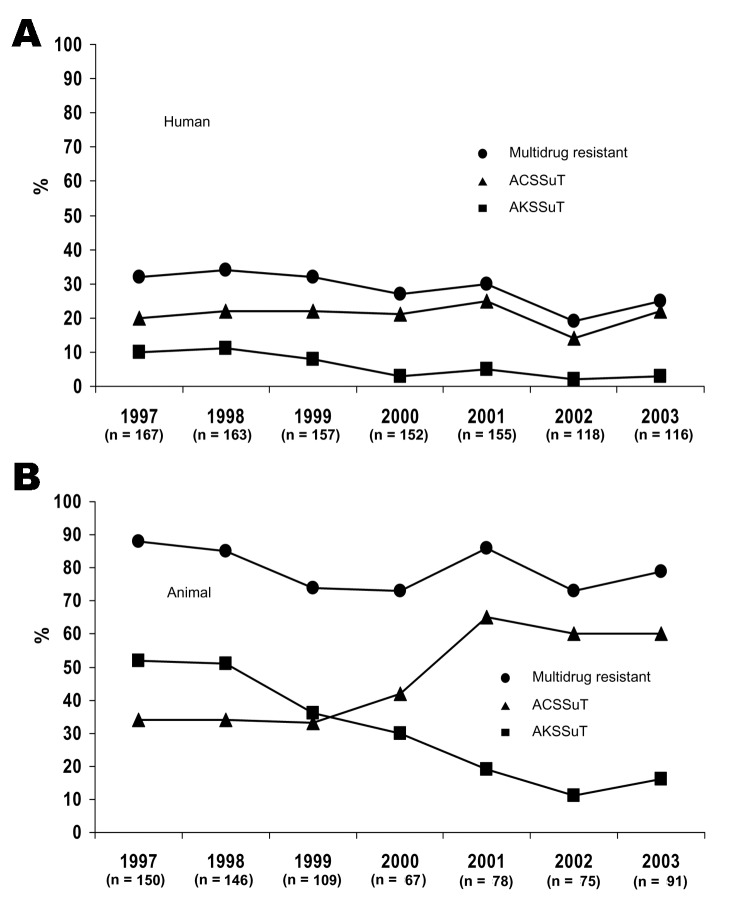
Percentage of Salmonella enterica serovar Typhimurium isolates from Minnesota humans (A) and animals (B) with multidrug resistance (i.e., resistance to >5 antimicrobial drugs), including resistance phenotypes (R-types) ACSSuT and AKSSuT, 1997–2003. A, ampicillin; C, chloramphenicol; K, kanamycin; S, streptomycin; Su, sulfisoxazole; T, tetracycline. R-type ACKSSuT is included as R-type ACSSuT but not AKSSuT.

Of the 1,028 *S*. Typhimurium isolates, 455 (44%) were resistant to >1 antimicrobial drug, and 296 (29%) were MDR (Table 1). Among MDR isolates, 217 (73%) were at least R-type ACSSuT, and 64 (22%) were at least AKSSuT (Table 2). The proportion of MDR isolates decreased from 32% in 1997 to 25% in 2003 (χ^2^ for linear trend 6.3, p = 0.01) (Figure 1). The proportion that were at least AKSSuT also decreased, from 10% in 1997 to 3% in 2003 (χ^2^ for linear trend 17.7, p<0.001).

**Table 2:** Distribution of human and animal isolate resistance phenotypes in PFGE clonal groups, Minnesota 1997–2003*

Clonal group†		No. isolates by resistance phenotype					
		At least ACSSuT‡	At least AKSSuT‡	At least ACKSSuT	Other resistance phenotypes	Pansusceptible isolates	Total
Human isolates							
	Clonal group A	169	1	12	27	8	217
	Clonal group B	1	51	1	23	5	81
	Other	22	12	12	124	560	730
	Total	192	64	25	174	573	1,028
Animal isolates							
	Clonal group A	182	3	67	10	2	264
	Clonal group B	1	227	21	27	2	278
	Other	21	20	23	38	72	174
	Total	204	250	111	75	76	716

*PFGE, pulsed-field gel electrophoresis; A, ampicillin; C, chloramphenicol; K, kanamycin; S, streptomycin; Su, sulfisoxazole; T, tetracycline.

†Clonal groups are composed of subtypes that are <3 bands different from PFGE subtype TM5b (clonal group A) or <3 bands different from subtype TM54 (clonal group B).

‡Does not include ACKSSuT.

Eighteen (1.8%) isolates were resistant to ceftriaxone; all were MDR (Table 1). Ceftriaxone resistance was more prevalent from 2000 to 2003 (2.8%) than from 1997 to 1999 (0.6%) (OR 4.6, 95% confidence interval [CI] 1.4–20.0, p = 0.008). Eleven (1.2%) isolates were resistant to nalidixic acid; all were MDR. Nalidixic acid resistance was more prevalent from 2000 to 2003 (1.8%) than from 1997 to 1999 (0.2%) (OR 9.2, 95% CI 1.5–200.8, p = 0.011). Fifty-one (5%) isolates were resistant to trimethoprim-sulfamethoxazole. Of these, 34 (67%) were MDR, including 20 (39%) that were at least R-type ACSSuT and 6 (12%) that were at least AKSSuT. Forty-three (4%) isolates were resistant to gentamicin; of these, 23 (53%) were MDR.

**Figure 2:**
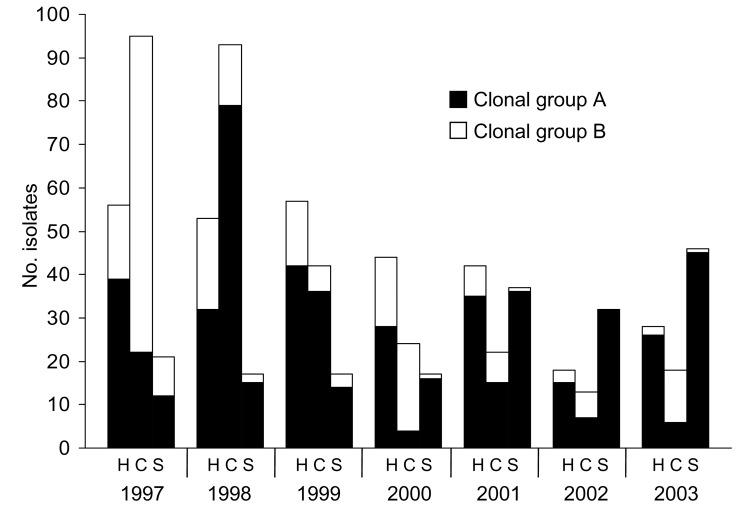
Distribution of Salmonella enterica serovar Typhimurium clonal group A pulsed-field gel electrophoresis (PFGE) subtypes and clonal group B PFGE subtypes among clinical isolates from humans and animals by species, Minnesota, 1997–2003. Clonal group A subtypes were <3 bands different from subtype TM5b by PFGE and were associated with resistance to ampicillin, chloramphenicol, streptomycin, sulfisoxazole, and tetracycline. Clonal group B PFGE subtypes were <3 bands different from subtype TM54 and were associated with resistance to ampicillin, kanamycin, streptomycin, sulfisoxazole, and tetracycline. H, C, and S indicate human, cattle, and swine isolates, respectively.

**Figure 3:**
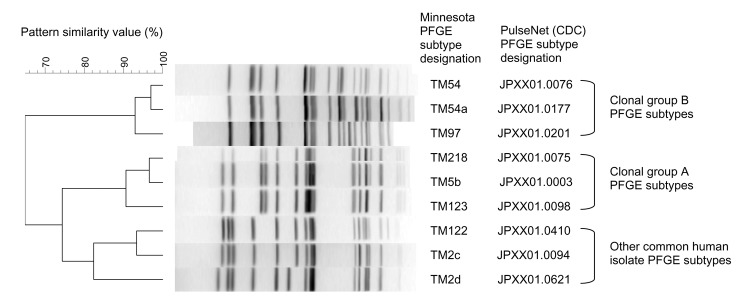
Pulsed-field gel electrophoresis (PFGE) patterns of common Salmonella enterica serovar Typhimurium subtypes observed among clinical isolates from humans and animals in Minnesota. The 3 clonal group B (CGB) PFGE subtypes represent the 3 most common CGB subtypes in animals and humans. The 3 clonal group A (CGA) PFGE subtypes represent the most common CGA subtypes in animals and humans. PulseNet designations are those used in the PulseNet national database of the Centers for Disease Control and Prevention (CDC).

We identified 271 unique PFGE subtypes among the 1,028 human *S*. Typhimurium isolates (median 63 subtypes/year, range 52–72). The 10 most common subtypes accounted for 509 (50%) isolates. CGA was composed of 31 PFGE subtypes. These subtypes accounted for 217 (21%) of all 1,028 human isolates, 188 (64%) of 296 MDR isolates, and 181 (83%) of 217 isolates that were at least R-type ACSSuT, including 12 isolates that were at least R-type ACKSSuT (Table 2, Figures 2 and 3).

CGB was composed of 20 subtypes and accounted for 81 (8%) of all 1,028 human isolates, 55 (19%) of 296 MDR isolates, and 51 (80%) of 64 isolates that were at least R-type AKSSuT (Table 2, Figures 2 and 3). The number of isolates with CGB subtypes decreased substantially from 2001 to 2003 (Figure 2).

### Animal Isolates

Overall, 640 (89%) of the 716 animal *S*. Typhimurium isolates were resistant to >1 antimicrobial drug, and 580 (81%) were MDR (Table 1). Of the 580 MDR isolates, 315 (54%) were at least ACSSuT, and 250 (43%) were at least AKSSuT (Table 2). The proportion of isolates that were at least ACSSuT increased over time (χ^2^ for linear trend 39.5, p<0.001). Conversely, the proportion that were at least AKSSuT decreased (χ^2^ for linear trend 71.7, p<0.001) (Figure 1).

Of the 358 cattle isolates, 205 (57%) were at least R-type AKSSuT, and 101 (28%) were at least ACSSuT. The decrease in cattle isolates over time reflected a decrease in the number that were at least AKSSuT (Figure 2). In addition, the proportion of cattle isolates that were at least AKSSuT decreased significantly over time (χ^2^ for linear trend 8.9, p = 0.003).

Of the 251 swine isolates, 180 (72%) were at least R-type ACSSuT, and 30 (12%) were at least AKSSuT. The increase in swine isolates over time reflected an increase in the number that were at least ACSSuT (Figure 2). In addition, the proportion of swine isolates that were at least ACSSuT increased significantly over time (χ^2^ for linear trend 25.4, p<0.001). Nine (24%) of 38 avian isolates, 19 (66%) of 29 equine isolates, and 15 (71%) of 21 feline isolates were MDR.

Twenty-five (3.5%) animal isolates were resistant to ceftriaxone. Ceftriaxone resistance was more prevalent from 2000 to 2003 (5.1%) than from 1997 to 1999 (2.2%) (OR 2.4, 95% CI 1.0–5.7, p = 0.035). Twelve ceftriaxone-resistant isolates were from cattle, and 10 were from swine. Four (0.6%) animal isolates were resistant to nalidixic acid, including 1 bovine isolate in 1997 and 3 turkey isolates in 2003. Eighty-one (11%) animal isolates were resistant to trimethoprim-sulfamethoxazole. Of these, 79 (98%) were MDR, and 62 (77%) were at least ACSSuT. Seventy-one (10%) animal isolates were resistant to gentamicin. Of these, 69 (97%) were MDR, and 44 (62%) were at least ACSSuT.

A total of 190 unique PFGE subtypes were identified among the 716 animal isolates (median 36 subtypes/year, range 31–47). Among animal isolates, CGA was composed of 48 PFGE subtypes. CGA accounted for 264 (37%) of all 716 animal isolates, 256 (44%) of 580 MDR isolates, and 249 (79%) of 315 isolates that were at least R-type ACSSuT, including 67 at least ACKSSuT isolates (Table 2, Figures 2 and 3). CGB was composed of 35 subtypes. CGB accounted for 278 (39%) of all 716 animal isolates, 250 (43%) of 580 MDR isolates, and 227 (91%) of 250 isolates that were at least R-type AKSSuT.

**Figure 4:**
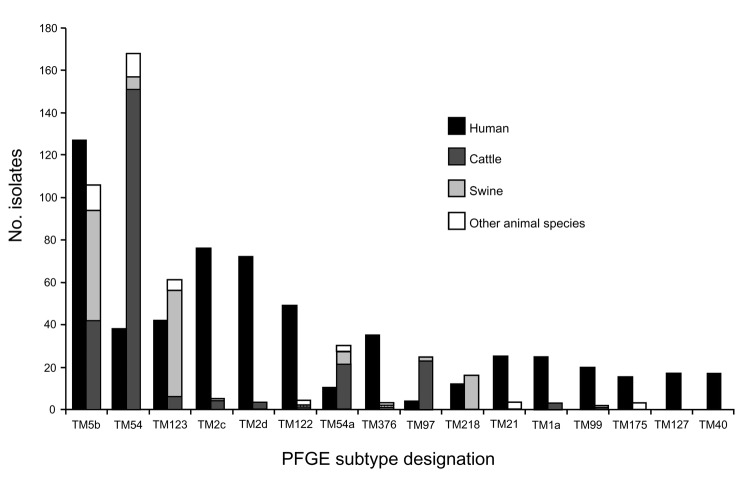
Frequency of pulsed-field gel electrophoresis (PFGE) subtypes that occurred >15 times among clinical human or animal Salmonella enterica serovar Typhimurium isolates in Minnesota, 1997–2003. Subtypes TM5b, TM123, and TM218 are part of clonal group A (subtypes <3 bands different from subtype TM5b). Subtypes TM54, TM54a, and TM97 are part of clonal group B (subtypes <3 bands different from subtype TM54).

Distribution of PFGE subtypes differed by species and year (Figures 2 and 4). CGB subtypes occurred predominantly in cattle and accounted for 67% of cattle isolates. As with AKSSuT isolates, CGB subtype isolates were numerous in cattle from 1997 to 1998, but the number dropped markedly in 2002 and 2003 (Figure 2). CGA subtype isolates increased in swine from 2000 to 2003 and substantially outnumbered CGA cattle isolates during those years. CGA isolates in cattle were most common from 1997 to 1998 and then declined to a relatively stable, low level (Figure 2).

Of 9 MDR avian isolates, 5 were in CGA and 1 was in CGB. Of 19 MDR equine isolates, 4 were in CGA and 5 were in CGB. Of 15 MDR feline isolates, 8 were in CGA and 6 were in CGB.

### Animal-Human Isolate Comparison

Combining the 1,028 human and 716 animal *S*. Typhimurium study isolates, 395 PFGE subtypes were identified. Sixty-six subtypes occurred both in animals and humans. These 66 subtypes represented 673 (65%) of human and 537 (75%) of animal isolates. Eighteen (27%) of shared subtypes were in CGA, and 12 (18%) were in CGB.

Combining the 296 MDR human isolates and the 580 MDR animal isolates, 183 PFGE subtypes were identified. Of these subtypes, 31 occurred both among human and animal MDR isolates. These 31 subtypes represented 237 (80%) human MDR isolates and 442 (76%) animal MDR isolates. Eighteen of the 31 shared MDR subtypes were in CGA, and 7 were in CGB. Of the 296 MDR human isolates, 177 (60%) had a CGA subtype that also occurred among MDR animal isolates, and 51 (17%) had a CGB subtype that also occurred among MDR animal isolates. Of the 296 MDR human isolates, 243 (82%) belonged to CGA (64%) or CGB (19%). Of the 580 MDR animal isolates, 506 (87%) belonged to CGA (44%) or CGB (43%).

The 6 most common individual subtypes in animals, all of which were in CGA or CGB (Figure 3), were represented among human isolates (Figure 4). TM5b, the second most common animal subtype, was the most common human subtype. TM54, the most common animal subtype, was sixth in humans. TM123 was the third most common animal subtype and fifth in humans (Figure 4).

## Discussion

This study provides a comprehensive comparison of clinical human and animal *S*. Typhimurium isolates from the same area. Overall, 29% of human *S*. Typhimurium isolates in Minnesota were MDR. Isolates with at least R-types ACSSuT or AKSSuT made up almost all (95%) of MDR *S*. Typhimurium in humans. Resistance phenotypes that were at least ACSSuT predominated. The level of multidrug resistance in human isolates decreased from 1997 to 2003, corresponding to a decrease in R-type AKSSuT isolates. Resistance to at least ACSSuT was stable over time. The level of multidrug resistance observed in human isolates in Minnesota was slightly lower than that observed through the National Antimicrobial Resistance Monitoring System (NARMS) through 2002; however, multidrug resistance trends for *S*. Typhimurium generally paralleled NARMS findings (*4**,**23*).

Increasing resistance to ceftriaxone documented in human isolates in Minnesota indicated that ceftriaxone resistance continues to emerge in *S*. Typhimurium in the United States (*13**,**24*). The 1.8% resistance to nalidixic acid observed in human isolates from 2000 to 2003 was not substantially higher than the 1% resistance among NARMS isolates from 2000 to 2002 (*23*) but was significantly higher than that seen in our isolates from 1997 to 1999. Most of the isolates that were resistant to both ceftriaxone and nalidixic acid were from 2000 or later. Resistance to these antimicrobial agents, as well as gentamicin and trimethoprim-sulfamethoxazole, frequently occurred in isolates that were also resistant to >5 other antimicrobial drugs; this finding was true for all isolates that were resistant to ceftriaxone or nalidixic acid. Resistance to these clinically important antimicrobial drugs was associated most frequently with ACSSuT resistance rather than AKSSuT resistance.

The increasing resistance to ceftriaxone and nalidixic acid (an elementary quinolone) is of concern because extended-spectrum cephalosporins and fluoroquinolones are needed to treat serious *Salmonella* infections. Recent experiences in Denmark have shown treatment failures and excess deaths associated with quinolone-resistant *S*. Typhimurium (*8**,**9*). The addition of resistance to clinically useful antimicrobial drugs to already-pentaresistant R-types is added cause for concern because pentaresistant *S*. Typhimurium strains are more likely to cause infection (*5*) and adverse health outcomes (*6**,**7*) than drug-susceptible strains.

Despite the overall diversity observed among *S*. Typhimurium isolates by PFGE, human MDR isolates were highly clonal. Even when a relatively stringent definition of a clonal group (<3-band difference) was used, >80% of human MDR isolates composed 2 clonal groups. CGA isolates were characterized by ACSSuT resistance and represented most human MDR isolates. Of isolates from this study that were previously phage typed, those in CGA have all been in the DT104 complex (*12**,**25**,**26*). The clonal nature of ACSSuT/DT104 *S*. Typhimurium in the United States has been well documented (*20**,**27*).

CGB isolates were characterized by AKSSuT resistance. This group accounted for 19% of human MDR isolates overall but was more prevalent early in the study, after which a marked decline occurred. As with the ACSSuT/DT104 complex, AKSSuT isolates appear to be largely clonal in nature.

Most *S*. Typhimurium isolates from clinically ill animals in Minnesota were MDR, which emphasizes that MDR strains are prevalent animal pathogens (*10*). High resistance levels occurred in all species, throughout the state, and during the entire study period. As with humans, most MDR animal isolates were in either the CGA/ACSSuT (DT104) or CGB/AKSSuT clonal groups. PFGE subtypes found among human and animal MDR isolates were remarkably similar. This similarity is striking considering that Minnesota residents may be exposed to *S*. Typhimurium during travel or from food produced outside Minnesota.

Among animals, the CGB/AKSSuT clonal group was most common in cattle. The sharp decrease in CGB isolates in cattle was mirrored by a similar decrease in humans. The cause of this decrease in cattle is not known. The CGA/ACSSuT clonal group was distributed more evenly among all animal species but became more common in swine over time. The cause for the increase in swine CGA/ACSSuT isolates is not known.

MDR *S*. Typhimurium strains similar to those from our study have been recovered from food animals and retail meat products by other investigators, and multiple MDR *S*. Typhimurium outbreaks caused by foods of animal origin or animal contact have been documented (*8**,**10**,**11**,**13**–**16**,**28**,**29*). Our data provide additional evidence that food animals are the primary reservoir of MDR *S*. Typhimurium for humans; MDR *S*. Typhimurium that belong to CGA or CGB were documented in cattle or swine herds on hundreds of farms throughout Minnesota. Testing isolates with additional genetic subtyping methods and identifying resistance determinants would help further characterize the relationship between animal and human isolates (*22**,**30*). In addition, data on use of antimicrobial drugs in animal production (which are currently unavailable in the United States because requirements are lacking) would be helpful in assessing this issue.

Although the number of isolates was relatively small, the level of multidrug resistance was high in both cat and horse isolates. CGA/ACSSuT and CGB/AKSSuT isolates were observed in both species. The importance of these infections in companion animals has been demonstrated by recent MDR *S*. Typhimurium outbreaks in humans associated with small animal veterinary facilities, including a Minnesota outbreak of CGA/ACSSuT DT104 infections in persons who adopted infected kittens from a humane society (*12*).

The source of animal isolates for our study is a limitation in that *Salmonella* isolates from clinically ill animals overstate the level of antimicrobial resistance observed in isolates from healthy animals; therefore, strains from ill animals are not representative of strains carried by animals at slaughter (*31**,**32*). However, when we have evaluated *S*. Typhimurium isolates from other studies, the most prominent CGA and CGB subtypes from our study also have been found in healthy food animals or their environments. For example, TM5b and TM123 isolates were recovered from healthy, market-ready pigs at slaughter (J.B. Bender, unpub. data). Subtypes TM5b, TM123, and TM54 were represented among poultry isolates evaluated by Rajashekara et al. (*28*). In a study of *Salmonella* isolates on dairy farms in 4 states, including Minnesota, subtypes TM5b and TM54 were recovered from healthy dairy cows or environmental samples (*33*). Finally, MDR *S*. Typhimurium is present in the retail meat supply; in a recent study, almost all strains of *S*. Typhimurium recovered from ground meat (pork and chicken) were MDR phage types DT104 or DT208 (*29*).

Another limitation of our study was the underrepresentation of poultry isolates. Minnesota is a leading poultry producer; however, most poultry diagnostics are conducted by the Minnesota Poultry Testing Laboratory. This laboratory has documented DT104 in Minnesota poultry (*28*). In our study, 3 of 4 nalidixic acid–resistant animal isolates were from turkeys, even though very few turkey isolates were tested. The role of poultry as a potential reservoir for MDR *S*. Typhimurium, including nalidixic acid–resistant strains, should be more thoroughly evaluated.

We agree with other investigators that the emergence of multidrug resistance in *S*. Typhimurium is associated with the widespread dissemination of clonal groups (*27**,**34*). The changing trends of MDR *S*. Typhimurium in cattle versus swine observed in our study and the presence of MDR strains in poultry indicate that more study of individual subtypes and resistance determinants (including specific mobile genetic elements) is required to understand the movement of these strains within and between animal species. Improved biosecurity practices to interrupt dissemination are undoubtedly the key in controlling these strains (*27*).

The potential role of the selection pressure of antimicrobial drugs used in animal agriculture in the dissemination of MDR *S*. Typhimurium clonal groups must be considered. The ability of MDR *S*. Typhimurium strains to accumulate additional resistances allows them to survive under a wide range of conditions when antimicrobial agents are used. Use of antimicrobial drugs to which MDR *S*. Typhimurium strains are already resistant may increase the number of animals infected with these strains and the number of animals that manifest clinical illness. This use is inherently likely to contribute to increased dissemination, both within and between farms. Thus, we encourage the judicious use of all antimicrobial drugs in animals as well as in humans. In particular, the recommendation (*19*) that nonessential uses of specific antimicrobial drugs in food animals should be eliminated (e.g., the use of tetracyclines and penicillins for growth promotion and feed efficiency) has merit. MDR *S*. Typhimurium strains are serious pathogens in food animals and humans. Restricting conditions that favor their dissemination should return the benefits of reduced incidence and severity of *S*. Typhimurium infections in both animals and humans.
